# Seasonal distribution of fish larvae in mangrove-seagrass seascapes of Zanzibar (Tanzania)

**DOI:** 10.1038/s41598-022-07931-9

**Published:** 2022-03-09

**Authors:** Barnabas Tarimo, Monika Winder, Matern S. P. Mtolera, Christopher A. Muhando, Martin Gullström

**Affiliations:** 1 Department of Ecology, Environment and Plant Sciences, Stockholm University, Stockholm, Sweden; 2 Institute of Marine Sciences, University of Dar Es Salaam, Zanzibar, Tanzania; 3 School of Natural Sciences, Technology and Environmental Studies, Södertörn University, Huddinge, Sweden

**Keywords:** Ecology, Ecology

## Abstract

Fish larvae supply in nearshore vegetated habitats, such as seagrass meadows and mangroves, contributes significantly to sustainable fish stocks. Yet, little information is available on distribution patterns of fish larvae in mangrove and seagrass habitats of the western Indian Ocean. The present study investigated the abundance, diversity and assemblage composition of fish larvae in mangrove creeks, inshore seagrass meadows (located adjacent to mangroves) and nearshore seagrass meadows (located in-between mangroves and coral reefs) in two coastal seascapes of Zanzibar (Tanzania) across seasons for 1 year. The highest mean abundances of fish larvae were recorded in mangrove creeks, while nearshore- and inshore seagrass meadows showed similar mean abundance levels. Generally, fish larvae representing 42 families were identified, with the fourteen most abundant families comprising 83% of all specimens. Fish larvae communities were dominated by specimens of the postflexion growth stage in all habitats, except in mangrove creeks in one of the two seascapes (i.e. Chwaka Bay) from April through June when abundances of the preflexion and very small-sized individuals were exceptionally high. Slightly higher fish larvae abundances were observed in mangroves during the southeast monsoon compared to the northeast monsoon, and there were also differences across months within monsoon periods for all three habitats studied. Assemblage composition of larvae did, however, not vary significantly in time or space. Our findings suggest that mangroves and seagrass meadows are highly linked shallow-water habitats with high retention of fish larvae contributing to similarity in assemblage compositions across shallow coastal seascapes. Conservation and management efforts should prioritize connected shallow-water seascapes for protection of fish larvae and to uphold sustainable coastal fisheries.

## Introduction

Shallow-water marine habitats are among the most productive areas on earth, providing numerous important ecological and economic services, such as fisheries production^1–4^. In tropical coastal seascapes, mangroves and seagrass meadows are commonly the dominating shallow-water habitats and essential habitats for fish^5,6^, supporting a high abundance and diversity of fish at different life stages^7^. Fish distribution patterns in coastal seascapes clearly indicate migration between mangroves and seagrass meadows as well as the usage of these habitats by a large diversity of fish species^8^. Numerous fish species use these vegetated coastal habitats also as nursery and spawning grounds as the structural complexity provides shelter and avoidance of predation^9^, while calm water in protected environments, such as mangroves, are suitable for fish larvae to settle with often high plankton prey availability^10^. Consequently, these habitats harbor high abundance of fish larvae^11–13^. However, environmental conditions may change over the season and together with species life history affect spatiotemporal patterns of fish larvae distribution^14^. Yet, seasonal dynamics of fish larvae abundance and diversity are seldom studied in tropical coastal systems. This knowledge is critical to determine variations in recruitment of marine fish species based on the differences in larval settlement versus post-settlement mortality^15^, and to determine spawning seasons of fish species^16^, which is useful information for ecosystem management^17^.

While fish at early growth stages, particularly up to the preflexion stage, are expected to drift with currents, behavioural adaptations, such as diel vertical movement and ontogenetic habitat utilization, may enable larvae to maintain in certain habitats as larval development progresses^18,19^. At the late larval (postflexion) stage, fish can have a directional swimming of up to 2 cm/min and strongly use taxa-specific environmental cues to sense and move towards suitable habitats^20–23^, where they settle as juveniles^19,24,25^. Thus, the nature of a coastal area, such as open or protected from the ocean or tidal dynamics, affects the abundance and assemblage composition of fish larvae in shallow coastal areas^26–28^. In addition, fish larvae distribution is influenced by spawning stock, spawning aggregations and other mechanisms that promote reproductive success, which in turn affects the chance of fish larvae survival, settlement and recruitment^29–31^. In tropical waters with low variation in biophysical variables, seasonal dynamics are mainly related to adult fish spawning behaviour or larval survival (e.g.^32^). As a result, a detailed description of the spatiotemporal distribution pattern of fish larvae in nearshore habitats is not only contextualized fundamental information for ecologists but also of high relevance for efficient conservation and management of coastal fisheries resources^33,34^.

In tropical coastal seascapes of the western Indian Ocean, spatial and temporal distribution patterns of fish larvae are scarcely explored^35^, which limits the knowledge about fish spawning location, timing and duration (e.g.^36^). Additionally, insufficient knowledge in fish larvae distribution patterns limits the information in dispersal patterns and source-sink relationships of fish larvae in their habitats^37,38^, which may hinder the conservation practices^39^. A few studies have focused on spatial variability of fish larvae in coastal marine habitats of this region. For instance, Little et al.^40^ studied fish larvae distribution at different sites of a Kenyan mangrove creek and found a decreasing larval abundance (of non-resident species in particular) and diversity along a gradient from the mouth towards the upper part of the creek system. Hedberg et al.^41^ reported little differences between sites and among habitats (open waters, seagrass meadows and coral reefs) in coastal East Africa, with most fish larvae families occurring in all three habitats without preference. In this region, mangroves and seagrass meadows exist as highly connected habitat within the coastal seascape, with mangroves close to the land, followed by seagrass meadows and coral reefs further offshore^42^. Nutrient cycling, larval export and migratory fauna movements connect these habitats (i.e. mangroves, seagrass meadows and coral reefs). Mobile organisms, such as fish, relocate between these habitats for a variety of reasons, including feeding, spawning, seasonal migrations, and ontogenetic movements^43,44^. Habitat connectivity can be driven by water movements, such as tidal regimes and currents, which help to connect different systems by promoting the export of larvae and plankton from one environment to another^45^. Particularly, seasonal changes in the direction of the monsoon winds may influence assemblages of plankton and fish larvae^33,46^. As a result of the lack of static borders and rather high seascape connectivity, shallow-water habitats in tropical coastal seascapes require a broad seascape approach to properly monitor, assess and conserve fish larvae populations^42,45^.

**Figure 1:**
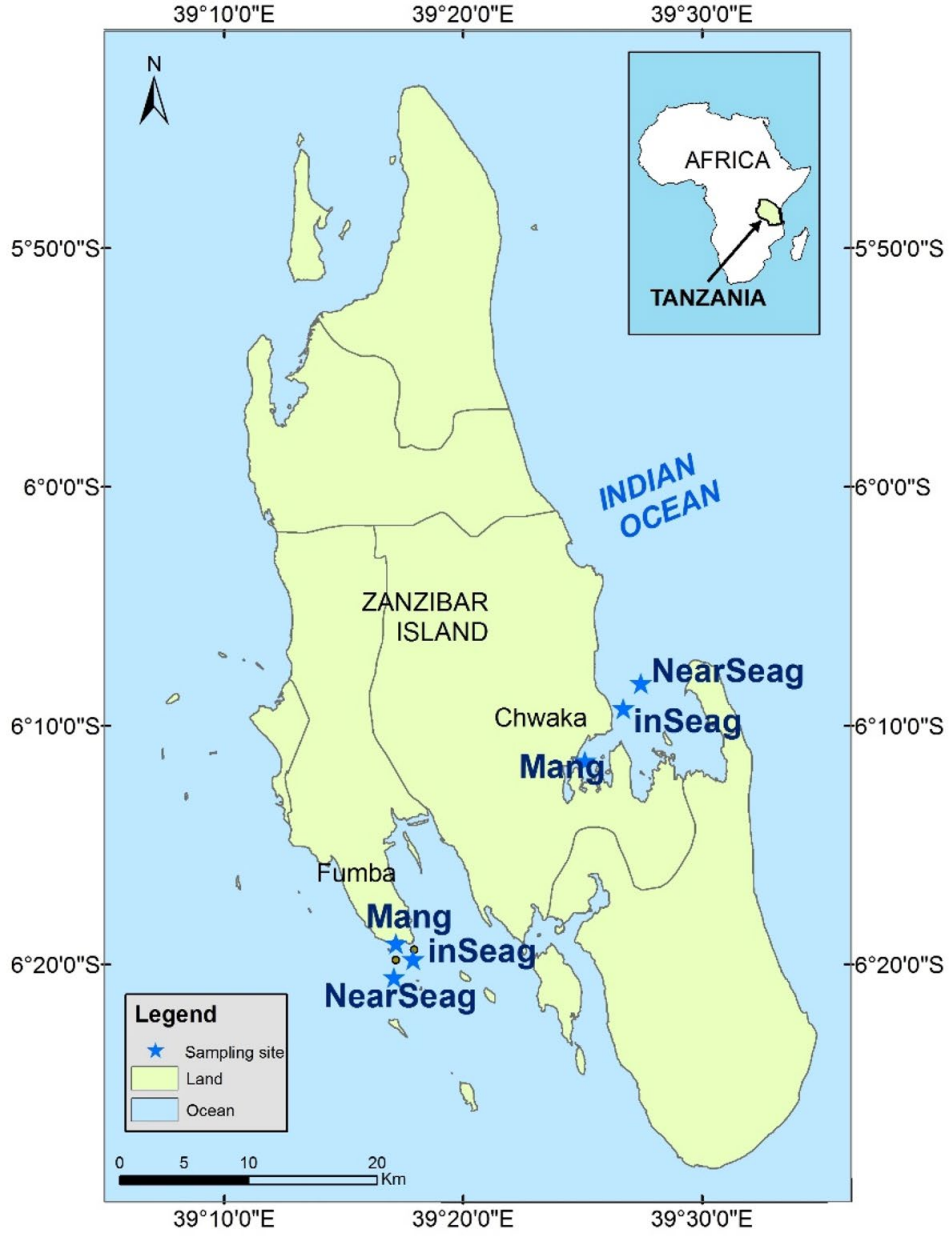
Map of Zanzibar showing locations of study sites, including mangrove creeks (Mang), inshore seagrass meadows (inSeag) (located adjacent to mangroves) and nearshore seagrass meadows (nearSeag) (located in-between mangroves and coral reefs) in Chwaka Bay (Chwaka) and Fumba on Zanzibar (Tanzania). Source: Institute of Marine Sciences Database, University of Dar es Salaam, Zanzibar (Tanzania).

In the present study, we investigated seasonal distribution patterns of fish larvae in mangrove creeks, inshore seagrass meadows (located adjacent to mangroves) and nearshore seagrass meadows (located in-between mangroves and coral reefs) in two tropical coastal seascapes of Zanzibar (Tanzania) in Eastern Africa. Sampling was conducted in Chwaka Bay, a semi-enclosed embayment, and Fumba, a non-estuarine coastal site (Fig. 1), monthly between January and December 2018. We hypothesized that the three habitats are strongly interlinked and harbour similar abundance, diversity and assemblage composition of fish larvae, and are linked to the monsoon seasons.

## Results

### Environmental parameters

In all three habitats of Chwaka Bay and Fumba, pH was relatively consistent throughout the year (Table S1). Water temperatures were generally higher during the NEM seasons (ranging from 29.9 to 31.5 °C) compared to the SEM seasons (ranging from 26.5 to 27.3 °C) (Table S1). Dissolved oxygen and salinity were higher during the NEM season than during the SEM season in all habitats in both sites (Table S1). Chlorophyll-a levels were substantially higher in mangroves of Chwaka Bay during both the NEM and SEM seasons compared to the levels in mangroves of Fumba and inshore and nearshore seagrass meadows in both sites (Table S1).

### General description of fish larvae assemblages and patterns of variability

**Figure 2:**
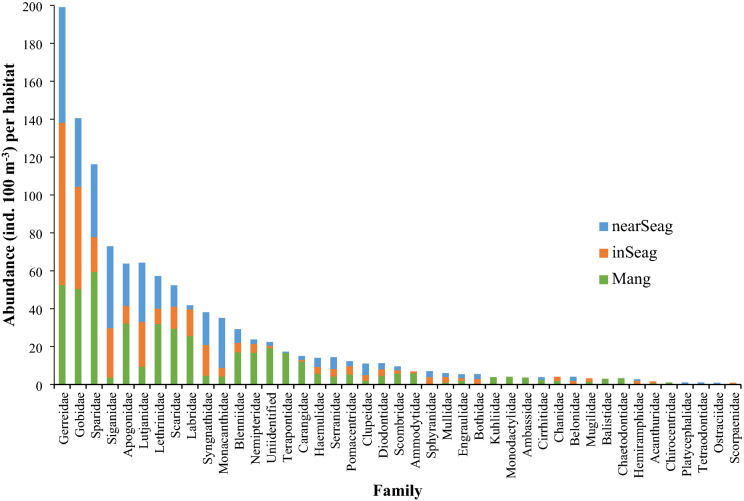
Mean abundance of fish larvae at family level in mangrove creeks (Mang), inshore seagrass meadows (inSeag) (located adjacent to mangroves) and nearshore seagrass meadows (nearSeag) (located in-between mangroves and coral reefs) in Chwaka Bay (Chwaka) and Fumba recorded for the whole sampling period (i.e. January–December 2018). The families are ranked from highest (left) to lowest (right) abundance.

A total of 2087 individuals of fish larvae of pelagic or pre-settlement phases were recorded throughout the sampling period, representing a total assemblage of 42 families (Table S2). The majority of fish larvae families (22) were observed to overlap in distribution across the three habitats (Table S2), while some few families were observed only in specific habitats at specific times. Six families were exclusively observed in mangrove creeks, one family in inshore seagrass meadows, and three families in nearshore seagrass meadows, while ten families overlapped between two habitats (Table S2). The 14 most abundant families comprised about 83% of the catch and included Gerreidae (18%), Gobiidae (12%), Sparidae (10%), Siganidae (6%), Apogonidae (6%), Lutjanidae (6%), Lethrinidae (5%), Scaridae (5%), Labridae (4%), Syngnathidae (3%), Monacanthidae (3%), Blenniidae (3%), Nemipteridae (2%) and Terapontidae (2%) (Fig. 2).

**Figure 3:**
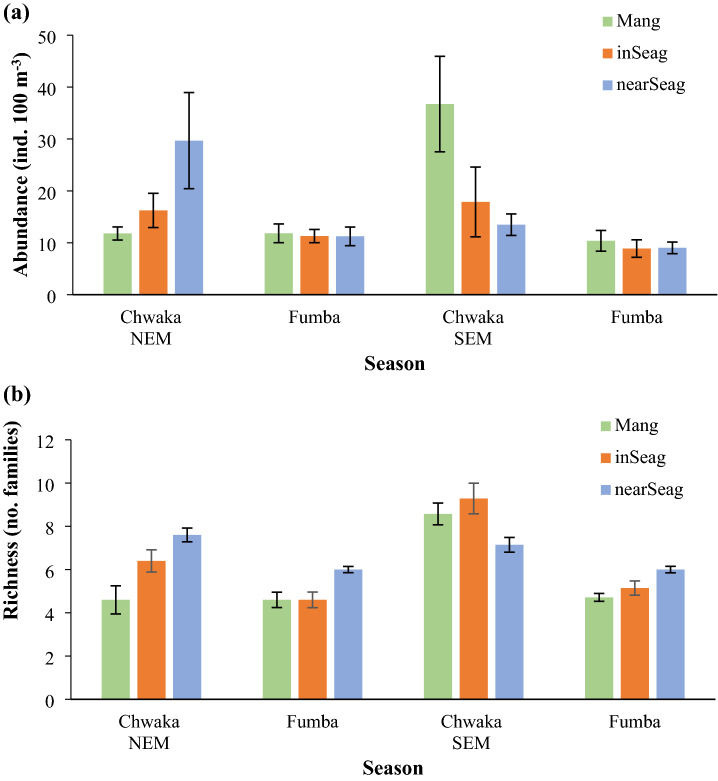
Mean abundance (± SE) of fish larvae (**a**) and mean number (± SE) of fish larvae families (**b**) in mangrove creeks (Mang), inshore seagrass meadows (inSeag) (located adjacent to mangroves) and nearshore seagrass meadows (nearSeag) (located in-between mangroves and coral reefs) in Chwaka Bay (Chwaka) and Fumba recorded for the NEM (November–March) and SEM (April–October) seasons across the sampling period (i.e. January–December 2018). NEM = northeast monsoon, SEM = southeast monsoon

Overall, the mean abundance of fish larvae was slightly (while not significantly; Table 1) higher in mangrove creeks compared to seagrass meadows, whereas inshore and nearshore seagrass meadows showed rather similar mean abundances (Fig. 3a). A three-way ANOVA on mean larvae abundance revealed statistical significance only between the two sites (with a higher mean abundance of fish larvae in Chwaka Bay compared to Fumba; Fig. 3a) and for an interaction between season and habitat as well as for an interaction between season, site and habitat (Table 1). Site-specific comparisons on habitat level showed that mangroves and nearshore seagrass meadows displayed higher mean fish larvae abundances in Chwaka Bay compared to the same respective habitat in Fumba (Holm–Sidak test at p < 0.01; Fig. 3a), which was also the case for inshore seagrass meadows (Fig. 3a), although the difference was not significant. In a three-way ANOVA on family richness of fish larvae, only site came out as a significant factor (Table 1) and which showed that the family richness was higher in Chwaka Bay compared to Fumba (Fig. 3b). A site-specific comparison revealed higher fish family richness within inshore seagrass meadows in Chwaka Bay compared to Fumba (Holm–Sidak test at p < 0.01; Fig. 3b), while no site-specific differences were seen for either mangroves or nearshore seagrass meadows (Fig. 3b).

**Table 1:** Summary of three-factor ANOVAs for fish larvae abundance and family richness.

Source of variation	Aundance				Family richness		
	df	MS	*F*	p	MS	*F*	p
Season	1	0.024	0.53	0.469	0.013	1.36	0.247
Site	1	0.966	21.06	** < 0.001**	0.097	10.08	**0.002**
Habitat	2	0.034	0.74	0.482	0.021	2.21	0.119
Season × Site	1	0.067	1.45	0.233	0.014	1044	0.234
Season × Habitat	2	0.196	4.28	**0.018**	0.004	0.42	0.659
Site × Habitat	2	0.015	0.33	0.721	0.004	0.38	0.683
Season × Site × Habitat	2	0.182	3.97	**0.024**	0.005	0.49	0.615
Residual	60	0.046			0.010		

Significant values (p < 0.05) are shown in bold.

### Seasonal variations of fish larvae abundance and family richness

**Figure 4:**
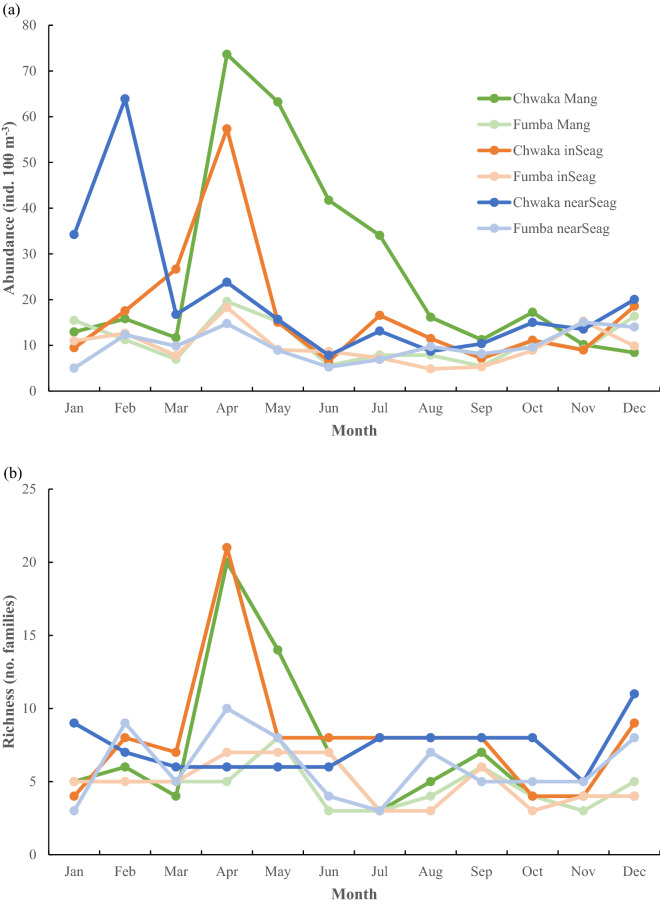
Mean abundance of fish larvae (**a**) and mean number of fish larvae families (**b**) in mangrove creeks (Mang), inshore seagrass meadows (inSeag) (located adjacent to mangroves) and nearshore seagrass meadows (nearSeag) (located in-between mangroves and coral reefs) in Chwaka Bay (Chwaka) and Fumba recorded for each month during the whole sampling period (i.e. January–December 2018).

The three-way ANOVAs showed no significant seasonal differences between SEM and NEM for either mean abundance or family richness of fish larvae (Table 1). The significant interactions (i.e. Season × Habitat and Season × Site × Habitat) on abundance data (Table 1), however, indicate that the seasonal fish larvae abundance patterns are not consistent in the two sites and/or in the three habitats (Fig. 4a). Specific seasonal comparisons on habitat level revealed that the mean fish larvae abundance in mangroves was higher during SEM compared to NEM (Holm–Sidak test at p < 0.05; Fig. 4a), whereas there were no significant monsoon-based differences in mean larvae abundance in any of the two seagrass habitats (Fig. 4a). Considering monthly differences across monsoon seasons, the abundance of fish larvae was clearly higher in mangrove creeks of Chwaka Bay during most of the SEM period (particularly April–July) compared to the NEM period, with the highest peak in April (Fig. 4a). There were also a few months in the NEM period with high peaks in abundance of fish larvae in inshore seagrass meadows (March and particularly April) and nearshore seagrass meadows (January and particularly February) (Fig. 4a). The fish larvae family richness varied slightly across monsoon seasons in the two sites and in the different habitats, with pronounced peaks only observed in mangrove creeks (particularly in April but also in May) and inshore seagrass meadows (April) of Chwaka Bay (Fig. 4b, Fig. S1). Seven fish larvae families were observed almost throughout the year in all three habitats, including Gerreidae, Sparidae, Gobiidae, Apogonidae, Siganidae, Lutjanidae and Syngnathidae (Fig. S1).

### Fish larvae assemblage compositions

**Figure 5:**
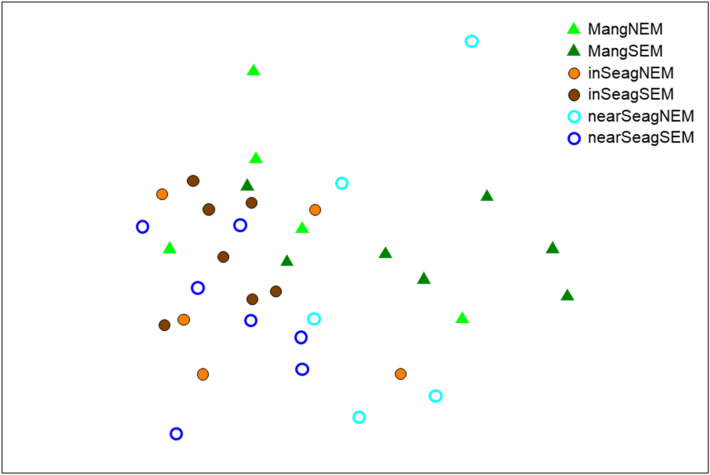
Non-parametric multidimensional scaling (nMDS) ordination of assemblage structure of the postflexion development stage of fish larvae in mangrove creeks (Mang), inshore seagrass meadows (inSeag) (located adjacent to mangroves) and nearshore seagrass meadows (nearSeag) (located in-between mangroves and coral reefs) in the southeast monsoon (SEM) period (April–October) and the northeast monsoon (NEM) period (November–March) on pooled data from Chwaka Bay and Fumba (Zanzibar, Tanzania) recorded for the whole sampling period (i.e. January–December 2018). The plot is based on square-root-transformed density data. The statistical stress is 0.20.

Abundance-based ANOSIM assessments of fish larvae revealed no clear differences in assemblage structure across months, between monsoon seasons, among habitats, between sites or for any combinations of these factors (Global R = − 0.075 to 0.253). The ANOSIM tests on postflexion fish larvae also showed few discernible separations in assemblage structure (Global R = − 0.072 to 0.234). Nevertheless, we found clear separations in assemblage structure of postflexion larvae between mangrove creeks and either of the two seagrass habitats (Mang vs. inSeag: R = 0.38, p = 0.001; Mang vs. nearSeag: R = 0.37, p = 0.003), while not between inshore and nearshore seagrass meadows, neither between monsoon seasons (visualized in Fig. 5).

### Growth stages and size composition

**Figure 6:**
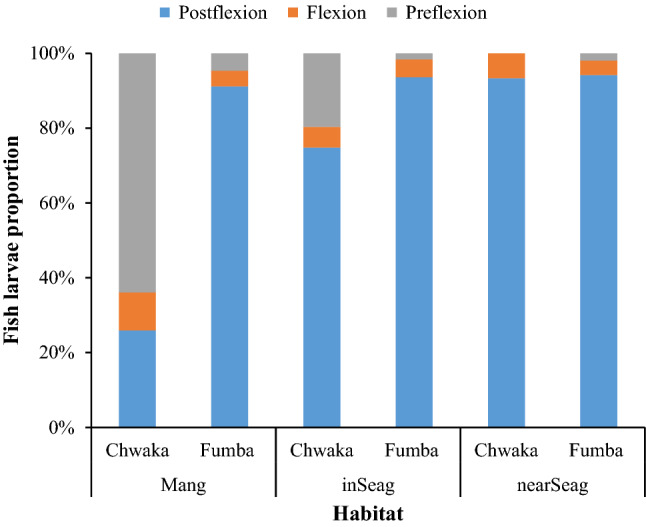
Proportion (%) of fish larvae in the different development stages (postflexion, flexion and preflexion) recorded in mangrove creeks (Mang), inshore seagrass meadows (inSeag) and nearshore seagrass meadows (nearSeag) in Chwaka Bay (Chwaka) and Fumba.

The majority of fish larvae (58%) were in the postflexion growth stage, while about 34% and 8% were in the preflexion and flexion development stages, respectively. Most of the fish larvae families were, however, composed of a mixture of individuals at all development stages. Mangrove creeks appeared to retain a larger proportion of very early life stages of fish in terms of size (length) and growth stage development compared to the two seagrass habitats (Fig. 6, Fig. S2). The fish larvae assemblages in mangrove creeks were dominated by small-sized specimens of less than 2 mm from April to June, which was particularly seen in Sparidae, Siganidae, Apogonidae, Lethrinidae, Scaridae and Labridae (Fig. S2). In comparison, mangrove creeks in Chwaka Bay appeared to support a much higher proportion of preflexion fishes (64%) compared to Fumba, which in contrast supported a high number of postflexion fishes (> 90%) (Fig. 6).

## Discussion

This study investigated the seasonal distribution patterns of fish larvae in mangrove-seagrass seascapes of Zanzibar (Tanzania) in the western Indian Ocean and revealed variability in abundance and family richness across the different seasons and months, while there were no distinct seasonal patterns of fish larvae assemblage composition in the studied mangrove creeks and seagrass meadows. These findings suggest that many fish larvae at pre-settlement stages are drifted and distributed stochastically within mangrove and seagrass habitats of this shallow coastal environment and therefore are not clearly determined by habitat characteristics in terms of vegetation type and coverage, seagrass shoot density and seagrass canopy height. Rather, other factors might be of importance. For instance, tidal current dynamics has been reported a strong driver of fish larvae abundance and assemblage composition in tropical regions worldwide^26,47,48^. In addition, seasonal distribution of fish larvae in shallow tropical coastal waters is commonly related to seasonal weather phenomena brought by monsoon winds (most pronounced in the upper layer of the water column)^49^ and contemporary oceanographic conditions^50–52^. Moreover, differences in abundance and family richness of larvae may have been influenced by spawning strategies and periods, recruitment success and abundance of adult fish stocks in the near surrounding^53,54^, but it is still unclear how these factors affect the overall fish larvae community composition in the habitats investigated. Another possible reason for the observed results may be high adult site fidelity, which is when fishes return to the same location on a regular basis, thereby contributing to the similarity of assemblages of their larval offspring^55,56^. Movement of adults and larvae, species-specific home range sizes^57,58^ and pelagic larval duration^59^ may have influenced distribution patterns of fish larvae, as the majority of adults (parents) of the fish larvae families observed in this study appear to have restricted home range sizes (a few km at most), in comparison to home range sizes reported elsewhere (see e.g.^60^). A small home range size can influence the movement of adult fishes and finally their offspring (larvae) that may only move between neighbouring habitats covering similar ecological functions, such as between mangroves and seagrass meadows^61^. According to Green et al.^60^, some important fish families in coastal shallow-water habitats, also encountered in this study, appear to have small home range sizes, including Syngnathidae, Pomacentridae, Chaetodontidae, Labridae, Lutjanidae, Serranidae, Scaridae, Mullidae and others. These are as opposed to species with long dispersal potential (pelagic larval duration), which may not have a significant impact on fish larvae distribution in coastal areas^59^. As a result, this could be one of the reasons why assemblage compositions of larvae did not differ significantly among habitats or across seasons (or even between the two sites) in this study. Within a lagoonal estuary, Able et al.^48^ also found a strong similarity in assemblage composition of fish larvae in interconnected shallow-water environments, which they attributed to regular movement of fish larvae driven by tidal exchanges between the estuary, the ocean and other nearby localities.

The majority of the fish larvae families in this study were found to overlap in distribution across the three habitats, which is an indication of strong habitat connectivity within the shallow-water seascapes^43^. Mangroves and seagrass meadows are interlinked because they share similar ecological functions to fishes (and other organisms), such as nursery grounds, foraging areas and shelter from predators^62^. The family richness of fish larvae (42 families) recorded in this study was within the range of those reported from other shallow coastal vegetated habitats of the western Indian Ocean region (varying between 29 and 51 fish larvae families), for instance tropical Tanzania, subtropical Mozambique^41^, coastal Kenya^16^, and nearshore coastal and estuarine environments of South Africa^27,63^. The predominance of fish larvae (83% of the catch) in the mangrove-seagrass seascapes of our study belong to families of Gerreidae, Gobiidae, Sparidae, Lutjanidae, Siganidae, Apogonidae, Lethrinidae, Scaridae, Labridae, Monacanthidae and Syngnathidae, and agrees well with what other studies have recorded in shallow-water vegetated habitats along the East African coast and in other Indo-Pacific areas^16,40,41,64–66^, which demonstrates the widespread phenomenon that many fish species commonly utilise aquatic vegetation as essential habitat during their larvae life-stages. The different types of fish larvae families found in these shallow-water seascapes suggest that their parents live in a variety of coastal ecosystems, such as mangroves, seagrass meadows, coral reefs and lagoonal estuaries, which could be related to the distance from the ocean^67^ and movement of adult fishes between coastal habitats (e.g.^24^).

The higher fish larvae abundance in mangroves in SEM compared to NEM and the observed monthly peaks in abundance and richness in both mangroves and seagrass meadows might correspond to high abundance and peaks in food production and spawning periods for some of the parent fish stocks^68^. A study by Ara et al.^69^ reported similar seasonal patterns of fish larvae abundance, richness and assemblage composition in mangrove and seagrass ecosystems of Malaysia. However, Mwaluma et al.^16^ reported higher abundance and richness of fish larvae in coastal Kenya during NEM compared to the SEM season. Seasonal differences in abundance and assemblage composition of fish larvae are therefore likely area-specific^70,71^, and might depend upon to food production and breeding season for some adult fish species.

We observed a weak but significant difference in assemblage composition of post-flexion larvae between mangrove creeks and the two seagrass meadow habitats (i.e. inshore and nearshore seagrass meadows) of Chwaka Bay, while in Fumba we did not find such differences in assemblage composition. As there was no seasonal influence, this suggests that the two sampling sites (Chwaka Bay and Fumba) are ecologically different and hence characterised by different biogeophysical driving forces. While Chwaka Bay is a partially enclosed lagoon, Fumba is a coastal area open towards the ocean and thus experiences a high variability in wave strength and circulation of currents. Due to the fact that postflexion fish larvae can move and maintain a position in a relatively calm area^20,72^, the reduced strength of currents due to a lagoon effect may help to maintain the distribution of postflexion fish larvae in Chwaka Bay habitats. This is in contrast to Fumba, where strong currents may accelerate the random drifting of postflexion larvae among the different shallow-water habitats (e.g.^73,74^). Additionally, the relatively high structural complexity of vegetation and calm environment in Chwaka Bay may have helped to retain early-stage fish larvae^13^, which might have contributed to the higher proportion of preflexion larvae observed in mangroves creeks of Chwaka Bay compared to Fumba. Meanwhile, the predominance of preflexion and small-sized fish larvae in April–June enlightens the peak spawning seasons of some parent fish stocks in the sampling areas (or near surroundings), which shows that preflexion and flexion fish larvae are likely distributed within vegetated habitats and retained in calm waters of mangrove creeks and inshore seagrass meadows. The overall high proportion of postflexion individuals observed in this study might partly be a result of the methodology used, i.e. the daytime horizontal surface hauling using plankton net in very shallow, tidally influenced nearshore areas, as smaller flexion and preflexion larvae may be found in deeper waters during daytime^75^. The mixture of individuals from all growth stages (including also 2% juveniles and adults sampled occasionally in the plankton net), however, indicates that the majority of fish larvae recorded in this study completed their pelagic phase in the same habitats, as shown by Pattrick & Strydom^63^.

## Conclusion

Our study characterized the seasonal distribution patterns of fish larvae in mangrove creeks and seagrass meadows in two coastal seascapes of the western Indian Ocean. The results showed differences in abundance and richness of fish larvae across the different months and between habitats, whereas assemblage composition did not show any distinguished seasonal or spatial patterns. Our findings suggest that mangroves and seagrass meadows are highly linked shallow-water habitats with high retention of fish larvae contributing to similarity in assemblage compositions across shallow coastal seascapes. Conservation and management efforts should be directed to prioritize connected shallow-water seascapes for protection of essential fish larvae habitats that together contribute to maintain healthy coastal fish stocks and sustainable coastal fisheries.

## Material and methods

### Study area description

Field sampling was conducted in Chwaka Bay and non-estuarine nearshore areas of Fumba on Zanzibar Island (Unguja), Tanzania, from January to December 2018 (Fig. 1). Chwaka Bay is a semi-enclosed embayment with a maximum average depth of 3.2 m at spring high tide and a total area of about 50 km^2^ at high water^76^. Mangroves are fringing the bay in the south with several creeks, while dense continued or disconnected seagrass meadows (commonly mixed but sometimes monospecific) of different complexity characterise large areas of the bay, which is bordered at the entrance by patch reefs^77,78^. Chwaka Bay is considered nursery ground for various fish species of economic and ecological importance^79,80^. In contrast to Chwaka Bay, the sampling area of Fumba is an open, non-estuarine environment in the coastal area of the Menai Bay Conservation Area (MBCA), where the main livelihood activities that surround the MBCA are fishing and agriculture^81^. The study area of Fumba is extensively covered by seagrass meadows, macroalgal belts, mangroves with small creeks and coral reefs with an average water depth of 10 m at high tide^82^. Fishing activities in both Chwaka Bay and Fumba are highly concentrated in nearshore areas with a subsequent pressure on the associated fish stocks^81^.

The southeast monsoon (SEM), lasting from April to October, drives the climatic conditions and is characterised by lower air temperatures, strong winds and rough sea, while the northeast monsoon (NEM) lasts from November to March and is characterised by higher air temperatures, lower wind speed and calm sea. There are two rainy seasons, including the long rain season from March to May and irregular short rains from September to November^49^. Mangroves in Chwaka Bay and Fumba are dominated by a muddy bottom substratum and turbid waters that fluctuate depending on runoff during different seasons, with average macroalgae coverage ranging from 3 to 29% (Table S3). *Thalassia hemprichii* dominated both inshore and nearshore seagrass meadows in the two study sites (Chwaka Bay and Fumba) (Table S3). All seagrass meadows, except inshore seagrass meadows in Fumba, were to some degree mixed with different seagrass species (i.e. *T. hemprichii*, *Enhalus acoroides*, *Cymodocea rotundata* and/or *Syringodium isoetifolium*) (Table S3). Calcareous algae (*Halimeda* spp.) as well as other macroalgae generally comprise a large part of seagrass meadows in Chwaka Bay^77^, while seagrass meadows of Fumba comprised macroalgae, such as *Gracilaria* spp. and *Chaetomorpha* spp.

### Habitat characterization, sampling of fish larvae and environmental parameters

In each seascape area (Chwaka Bay and Fumba), sampling sites were established (0.5–5 km apart in each habitat) in mangrove creeks (Mang), inshore seagrass meadows (inSeag) (located adjacent to mangroves) and nearshore seagrass meadows (nearSeag) (located in-between mangroves and coral reefs). Habitat characterizations in terms of habitat cover (%), seagrass canopy height (cm) and seagrass shoot density (number of shoots per m^−2^) were conducted along transects (100 m in length) at one occasion per month in January, March, July and September 2018 to capture potential differences across seasons. In each seagrass habitat, two transects were laid parallel to the shoreline, while in mangrove creeks, two transects were laid from upstream towards the mouth of the creek. Approximately 10 m apart, a quadrat of 0.5 m^2^ was thrown randomly five times in each transect. In each 0.5 m^2^ quadrat (n = 5), the percentage cover of seagrass, macroalgae and unvegetated area was quantified and seagrass species composition determined. A quadrat of 0.0625 m^2^, placed inside the 0.5 m^2^ quadrat frame, was used to assess seagrass canopy height and shoot density (n = 5). In addition, substrate bottom type was also determined as either rocky, muddy or sandy.

Sampling of fish larvae was performed in the three habitats (mangroves and the two seagrass habitats) during daytime (between 6:30 and 11:00 h) at high tide on a monthly basis from January through December 2018. The sampling was carried out using an ichthyoplankton net (500-μm in mesh size and a cod end of the same mesh size) with a mouth diameter of 0.5 m and a length of 2.5 m, fixed with a flowmeter in the mouth frame to determine the filtered volume of water. The plankton net was towed horizontally (at an average depth of 1 m) behind a small boat for 15 min (with a very low speed of approximately 1–1.5 knots, which is equivalent to 1.9–2.8 km per hr) and replicated two times in each habitat. After each tow, the fish larvae specimens were placed in sample bottles, quickly fixed with 75% ethanol solution and transported to the laboratory for further analysis. GPS coordinates were taken at each sampling site to be able to follow up the same locations throughout all sampling occasions. In situ water environmental parameters were measured (in triplicate) at the water surface in each habitat of the two sites (Chwaka Bay and Fumba) during every sampling occasion and included pH, water temperature, dissolved oxygen (DO) and salinity. Water temperature and pH were recorded in the field using a multiprobe pH meter with a temperature sensor (Model STX-3). A portable refractometer (HHTEC 4-i-1) was used to measure salinity, and a DO meter was used to measure dissolved oxygen (Extech 407510). Triplicate samples of 1.5 L of water for chlorophyll-a analysis was obtained using a water sampler at a depth of one meter, placed in a cold box and transported to the laboratory for chlorophyll-a (phytoplankton biomass) determination.

### Laboratory analyses

In the laboratory, fish larvae were separated from other zooplankton and debris using a stereomicroscope (Zeiss Stemi 508). Using the specialized identification guides by Jeyaseelan^83^, Mwaluma et al.^84^ and Leis and Carson-Ewart^85^, each fish specimen was taxonomically identified to family level and measured for total length (mm). The growth stage of each specimen was determined as either preflexion, flexion, postflexion or juvenile, and in the case of syngnathiforms (seahorses and pipefishes), they were determined as either larvae, juvenile or adult because they do not have differentiated growth stages as larvae. Distorted fish larvae or very small larvae at the egg yolk stage, which were difficult to identify, were grouped as unidentified. At the end of the sampling, some few late juveniles and adults (about 2% of the catch) were sampled occasionally in the plankton net, particularly in seagrass habitats, and mostly from the family Syngnathidae (pipefishes and seahorses), which are slow swimmers, and a few individuals from the families Serranidae, Scaridae and Apogonidae. Chlorophyll-a concentrations were measured spectrophotometrically in the laboratory using a Shimadzu UV–visible spectrophotometer, following protocols by Strickland and Parsons^86^.

### Data analysis

Before estimating the abundance of fish larvae (per 100 m^3^), all late juveniles (with all features of adult fish) and adult fishes were recorded and excluded from the catch. Habitat characteristics (i.e. habitat cover, seagrass canopy height and seagrass shoot density; Table S3) were compared among habitats, i.e. Mang, inSeag, and nearSeag, using one-way ANOVAs in SPSS version 20. Differences in fish larvae abundance and family richness were analysed using three-way model ANOVAs with Season (2 levels, fixed), Site (2 levels, fixed) and Habitat (3 levels, fixed) as explanatory factors. Prior to the ANOVA analyses, the assumption of homogeneity of variances was checked to discover if the data were normally distributed, and when it was heteroscedastic, the data were transformed using either log_10_ (for abundance data) or square root (for richness data) transformations. A posteriori multiple comparison tests were carried out on data from the significant interactions using the Holm-Sidak method. All analyses that concerned the three-way ANOVAs were performed in SigmaPlot version 14.0. Analysis of similarities (ANOSIM) was used to test for differences in assemblage structure across months, between monsoon seasons, among habitats, between sites and based on combinations of these factors. Since the postflexion growth stage comprised a large proportion of the catch, we did also separate multivariate analyses to test for differences in assemblage structure of fish larvae at this development stage. Patterns of similarities from one of the ANOSIM analyses were visualized using non-parametric multidimensional scaling (nMDS) based on Bray–curtis similarity index measures and calculated based on abundance data after square-root transformation. The multivariate analyses were performed using the PRIMER Software package^87^.

### Ethical statement

The study protocol was approved on the 23rd of January 2017 by the Department of Ecology Environment and Plant sciences (DEEP), Stockholm University, in collaboration with the Institute Postgraduate Studies Committee of the University of Dar es Salaam (UDSM) in compliance with the Tanzania Fisheries Act (2003) and the Wildlife Conservation Act (1974). We confirm that the study was undertaken with all procedures that minimize the pain and suffering, and improve animal welfare. The permit to sample and transport the larval fishes from the field to the laboratory was issued by the Ministry of Livestock and Fisheries and other local authorities for complying with the requirement of Fisheries Regulations (G.N. No. 308 of 2009).

### Consent to participate

The authors declare their participation in the study.

## Supplementary Information

Supplementary Information.

## Data Availability

Data from this research are available from the corresponding author on reasonable request.
