# Author Correction: Seasonal distribution of fish larvae in mangrove-seagrass seascapes of Zanzibar (Tanzania)

**DOI:** 10.1038/s41598-022-09088-x

**Published:** 2022-03-22

**Authors:** Barnabas Tarimo, Monika Winder, Matern S. P. Mtolera, Christopher A. Muhando, Martin Gullström

**Affiliations:** 1 Department of Ecology, Environment and Plant Sciences, Stockholm University, Stockholm, Sweden; 2 Institute of Marine Sciences, University of Dar Es Salaam, Zanzibar, Tanzania; 3 School of Natural Sciences, Technology and Environmental Studies, Södertörn University, Huddinge, Sweden

Correction to: *Scientific Reports* 10.1038/s41598-022-07931-9, published online 09 March 2022

The original version of this Article contained an error in Figure 4 where the unit, ‘m’, was omitted from the y-axis label in panel (a). The original Figure 4 and accompanying legend appear below.

**Figure 4:**
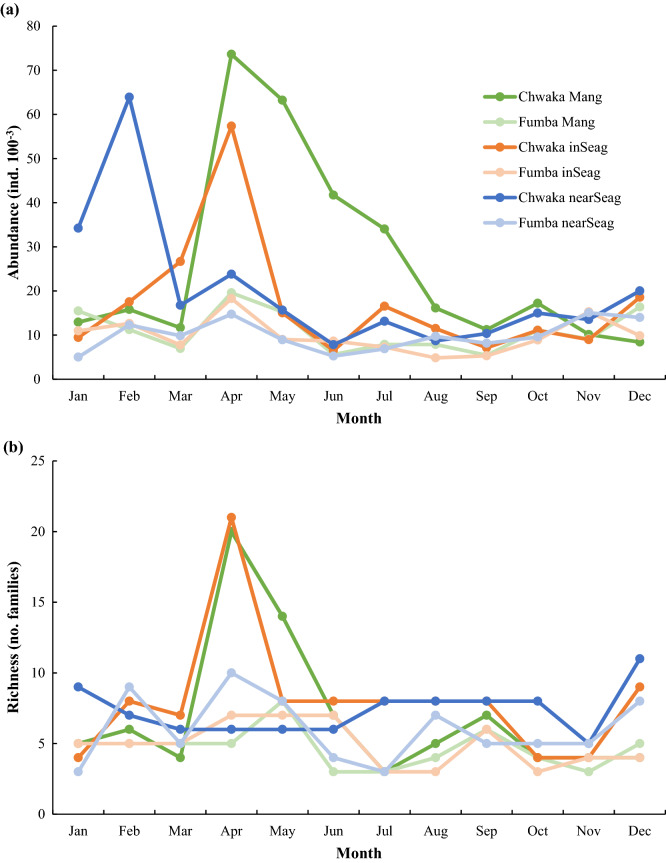
Mean abundance of fish larvae (**a**) and mean number of fish larvae families (**b**) in mangrove creeks (Mang), inshore seagrass meadows (inSeag) (located adjacent to mangroves) and nearshore seagrass meadows (nearSeag) (located in-between mangroves and coral reefs) in Chwaka Bay (Chwaka) and Fumba recorded for each month during the whole sampling period (i.e. January–December 2018).

The original Article has been corrected.

