# Supplementary material for: Seasonal distribution of fish larvae in mangrove-seagrass seascapes of Zanzibar (Tanzania)
Source: Sci Rep. 2022 Mar 9;12:4196. doi: 10.1038/s41598-022-07931-9 (PMC8907160; doi:10.1038/s41598-022-07931-9)
Supplement: Supplementary file 1 — Supplementary Information. [file 41598_2022_7931_MOESM1_ESM.docx]

**Supplementary information**

**Table S1.** In-situ environmental parameters (mean ± SE) recorded in mangrove creeks (Mang), inshore seagrass meadows (InSeag) and nearshore seagrass meadows (NearSeag) within Chwaka Bay (Chwaka) and Fumba on Zanzibar (Tanzania) during the NEM and SEM seasons. The environmental parameters include pH, water temperature (temp), dissolved oxygen (DO), salinity and Chlophyll-a (Chl-a). NEM = northeast monsoon, SEM = southeast monsoon

| **Habitat** | **Site** | **Season** | **pH** | **Temp (°C)** | **DO (mg/l)** | **Salinity** | | **Chl-a (mg/m^3^)** |
| --- | --- | --- | --- | --- | --- | --- | --- | --- |
| Mang | Chwaka | NEM | 8.1 ± 0.3 | 31.5 ± 0.8 | 9.4 ± 1.7 | 34.7 ± 0.9 | 2.7 ± 0.4 | |
| Mang | Chwaka | SEM | 8.2 ± 0.1 | 27.0 ± 0.4 | 5.0 ± 0.5 | 26.8 ± 3.8 | 2.4 ± 0.3 | |
| Mang | Fumba | NEM | 8.4 ± 0.1 | 31.4 ± 0.9 | 9.0 ± 1.6 | 35.2 ± 0.4 | 1.4 ± 0.1 | |
| Mang | Fumba | SEM | 8.3 ± 0.1 | 26.5 ± 0.4 | 5.7 ± 0.7 | 33.6 ± 0.8 | 1.3 ± 0.1 | |
| inSeag | Chwaka | NEM | 8.0 ± 0.2 | 31.0 ± 0.5 | 8.3 ± 0.7 | 35.5 ± 0.5 | 1.3 ± 0.1 | |
| inSeag | Chwaka | SEM | 8.5 ± 0.1 | 27.1 ± 0.3 | 7.1 ± 0.5 | 33.8 ± 0.9 | 1.4 ± 0.1 | |
| inSeag | Fumba | NEM | 8.3 ± 0.2 | 30.1 ± 0.4 | 7.9 ± 0.9 | 35.2 ± 0.1 | 1.3 ± 0.1 | |
| inSeag | Fumba | SEM | 8.5 ± 0.1 | 26.7 ± 0.3 | 6.8 ± 0.5 | 34.0 ± 0.5 | 1.2 ± 0.04 | |
| nearSeag | Chwaka | NEM | 7.7 ± 0.3 | 30.6 ± 0.2 | 7.8 ± 0.3 | 35.3 ± 0.9 | 1.2 ± 0.1 | |
| nearSeag | Chwaka | SEM | 8.5 ± 0.1 | 27.3 ± 0.3 | 7.6 ± 0.4 | 34.2 ± 0.9 | 1.3 ± 0.05 | |
| nearSeag | Fumba | NEM | 8.1 ± 0.2 | 29.9 ± 0.3 | 7.6 ± 0.4 | 35.0 ± 0.1 | 1.1 ± 0.1 | |
| nearSeag | Fumba | SEM | 8.5 ± 0.1 | 26.8 ± 0.3 | 6.9 ± 0.5 | 34.6 ± 0.2 | 1.1 ± 0.04 | |
|  |  |  |  |  |  |  |  | |

**Table S2.** Mean abundance and size range of each fish larvae family sampled in mangrove creeks (Mang), inshore seagrass meadows (inSeag), and nearshore seagrass meadows (nearSeag) within Chwaka Bay (Chwaka) and Fumba on Zanzibar (Tanzania) during the entire sampling period. (+) indicates presence of a particular fish family in a certain habitat, but with a contribution less than 2% of the total abundance of fish larvae, (-) indicates absence of a particular fish family in a certain habitat

| **Habitat** | **Mang** |  | **InSeag** |  | **NearSeag** |  |  |
| --- | --- | --- | --- | --- | --- | --- | --- |
| **Family** | **abundance**  **(n 100 m^–3)^** | **size range**  **(mm)** | **abundance (n 100 m^–3)^** | **size range (mm)** | **Abundance**  **(n 100 m–^3)^** | **size range (mm)** | **%** |
| Acanthuridae (Surgeonfishes) | + | 1.4 | + | 28.0 | - | - | 0.2 |
| Ambassidae (Glass Perchlets) | + | 1.7 - 11 | - | - | - | - | 0.3 |
| Ammodytidae (Sand Lances) | + | 1.2 - 4.6 | + | 1.4 - 1.7 | - | - | 0.6 |
| **Apogonidae (Cardinalfishes)** | 32.2 | 8.2 - 24 | 9.2 | 1.3 - 31 | 22.2 | 7.7 - 31 | **6.0** |
| Balistidae (Triggerfish) | + | 13 - 26 | - | - | - | - | 0.3 |
| Belonidae (Needlefishes, Longtoms) | - | - | + | 29 - 31 | + | 7.9 -10.6 | 0.4 |
| **Blenniidae (Combtooth Blennies)** | 17 | 1 - 21.7 | 5 | 1.2 - 20.6 | 7.1 | 7.8 - 28.1 | **3.0** |
| Bothidae (Lefteye Flounders) | - | - | + | 1.2 - 1.7 | + | 10.7 - 11.5 | 0.5 |
| Carangidae (Jacks, Pompanos, Trevallys) | + | 1 - 12.6 | + | 1 - 1.5 | + | 30 - 32 | 1.3 |
| Chaetodontidae (Butterflyfishes) | + | 11 - 20 | - | - | - | - | 0.3 |
| Chanidae (Milkfish) | + | 1 - 1.5 | + | 1.3 - 1.7 | - | - | 0.4 |
| Chirocentridae (Wolf herring) | + | 12 - 19 | - | - | - | - | 0.1 |
| Cirrhitidae (Hawkfishes) | + | 9 - 11 | - | - | + | 30 - 32 | 0.3 |
| Clupeidae (Herrings, Sardinellas, Sardines) | + | 6 - 9 | + | 8 - 20 | + | 11 - 20 | 1.0 |
| Diodontidae (Porcupinefishes) | + | 11 - 15 | + | 10 - 13.9 | + | 11 - 17 | 1.0 |
| Engraulidae (Anchovies) | + | 19 - 30 | + | 1.4 - 30 | + | 8.8 - 11.1 | 0.5 |
| **Gerreidae (Majjoras, Silver Biddies)** | 53 | 1.2 - 23 | 86 | 7 - 21 | 61 | 10 - 29 | **18.0** |
| **Gobiidae (Gobies, Mudskipper)** | 50 | 1 - 30.2 | 54 | 1 - 27 | 36 | 2.1 - 31.6 | **12.0** |
| Haemulidae (Grunts, Sweetlips) | + | 1.2 - 30 | + | 13 - 30 | + | 16 - 30 | 1.2 |
| Hemiramphidae (Halfbeaks, Garfishes) | - | - | + | 14.9 - 15.4 | + | 17.0 | 0.2 |
| Kuhlidae (Flagtail fishes, Aholeholes) | + | 11 - 19 | - | - | - | - | 0.4 |
| **Labridae (Wrasses)** | 25.6 | 1.8 - 8.3 | 14.1 | 1.1 - 18 | 2 | 22-29 | **4.0** |
| **Lethrinidae (Emperors and Big Eye Bream)** | 32 | 1- 17.9 | 9 | 3.3 - 27.6 | 17 | 10 - 29 | **5.0** |
| **Lutjanidae (Snappers and Fusiliers)** | 9.5 | 8.7 - 25.9 | 23.6 | 4.4 - 28.6 | 31.2 | 9.1 - 30.2 | **6.0** |
| **Monacanthidae (Filefishes, Leatherrjackets)** | 4 | 1 - 2.7 | 5 | 11.9 - 16.1 | 26 | 10 - 26.5 | **3.0** |
| Monodactilidae (Diamondfishes, Moonies) | + | 4 - 8.7 | - | - | - | - | 0.4 |
| Mugilidae (Mullets) | + | 7.0 | + | 1.2 - 1.5 | + | 5- 31 | 0.4 |
| Mullidae (Red Mullets, Goatfishes) | + | 7.6 - 5.9 | + | 19.9 - 20.4 | + | 9.3 - 11.1 | 0.5 |
| **Nemipteridae (Thread fin Breams, Monocle Breams)** | 16.8 | 1 - 30 | 4.7 | 1.1 - 10.1 | 2.3 | 7.8 - 25.1 | **2.0** |
| Ostraciidae (Boxfishes, Trunkfishes) | - | - | - | - | + | 15.0 | 0.1 |
| Platycephalidae (Flatheads) | - | - | - | - | + | 11.1 - 11.7 | 0.1 |
| Pomacentridae (Damselfishes) | + | 1.1 - 31 | + | 1.3 - 20.6 | + | 13 - 21.3 | 1.1 |
| **Scaridae (Parrotfishes)** | 29.5 | 1 - 7.3 | 11.6 | 1.2 - 25 | 11.2 | 8.3-24.1 | **5.0** |
| Scombridae (Tunas, Mackerels) | + | 1.2 - 3.6 | + | 1.1 - 1.8 | - | - | 0.8 |
| Scorpaenidae (Scorpionfishes, Stonefish) | - | - | + | 12.5 | - | - | 0.1 |
| Serranidae (Grouper, Sea bass) | + | 1 - 22 | + | 11.5 - 28 | + | 10.4 -25.1 | 1.3 |
| **Siganidae (Rabbitfishes)** | 3.7 | 1 - 25 | 26.1 | 11 - 31 | 43.1 | 9.1 - 33.4 | **6.0** |
| **Sparidae (Porgies, Breams)** | 59.4 | 1 - 23 | 18.3 | 6.2 - 21 | 38.4 | 6.7 - 27.1 | **10.0** |
| Sphyraenidae (Barracudas) | - | - | + | 20 - 26 | + | 20 - 28.1 | 0.6 |
| **Syngnathidae (Pipefishes, Seahorses)** | 4.7 | 7.7 - 20 | 16.3 | 3.1 - 20.1 | 17.3 | 5.1 - 20.1 | **3.0** |
| **Terapontidae (Grunters)** | 17.3 | 1 - 14.8 | - | - | + | 16.0 | **2.0** |
| Tetraodontidae (Pufferfishes, Swellfishes) | - | - | - | - | + | 15.8 | 0.1 |

**Table S3.** Habitat characteristics (mean ± SE) from mangrove creeks (Mang), inshore seagrass meadows (inSeag) and nearshore seagrass meadows (nearSeag) within Chwaka Bay (Chwaka) and Fumba on Zanzibar (Tanzania). Measurements include percent cover of a certain habitat (%), seagrass canopy height (cm) and seagrass shoot density (number of shoots, No. / m^-2^). The bolded values show significance between sites at p < 0.05. (-) indicates absence of the parameter in question

|  |  | Chwaka Bay | | | Fumba | | |
| --- | --- | --- | --- | --- | --- | --- | --- |
| Habitat | Measure | Mang | inSeag | nearSeag | Mang | inSeag | nearSeag |
| Unvegetated | % | 90 ± 1.4 | 10 ± 1.3 | 23± 5.9 | 70 ± 1.2 | 22 ± 1.7 | 20 ± 0.6 |
| Macroalgae | % | 3 ± 0.9 | 16 ± 0.5 | 17± 2.3 | 29 ± 0.2 | 21 ± 1.8 | 8 ± 0.4 |
| *T. hemprichii* (Th) | % | **-** | 67 ± 1.4 | 58 ± 4.8 | - | 57 ± 3.2 | 67 ± 0.8 |
|  | cm | **-** | 13 **±** 0.4 | 12 ^±^ 1.1 | - | 8 **±** 0.2 | 10 ± 0.4 |
|  | No. / m^2^ | **-** | **780 ± 25.6** | 679 ± 54.4 | - | **694 ± 5.6** | 850 ± 13.1 |
| *E. acoroide*s (Ea) | % | **-** | 23 ± 0.7 | 8 ± 4 | - | - | 2 ± 0.1 |
|  | cm | **-** | 19 ± 1.8 | 13 ± 4 | - | - | 5 ± 0.1 |
|  | No. / m^2^ | **-** | 118 ± 3 | 63 ± 1.3 | - | - | 67 ± 1.4 |
| *C. rotundata* (Cr) | % | **-** | 21 ± 1.8 | 12 ± 0.3 | - | - | 11 ± 2.2 |
|  | cm | **-** | 3 ± 0.8 | 5 ± 1.2 | - | - | - |
|  | No. / m^2^ | **-** | 36 ± 3.1 | 104 ± 2.7 | - | - | - |
| *S. isoetifolium* (Si) | % | **-** | - | 5 ± 1.3 | - | - | - |
|  | cm | **-** | - | 37± 1.8 | - | - | - |
|  | No. / m^2^ | **-** | - | 54 ± 13 | - | - | - |

**Figure S1.** Proportion (%) of larvae of fish families in different months recorded in (a) mangrove creeks, (b) inshore seagrass meadows, and (c) nearshore seagrass meadows within Chwaka Bay (Chwaka) and Fumba on Zanzibar (Tanzania) from January to December 2018.


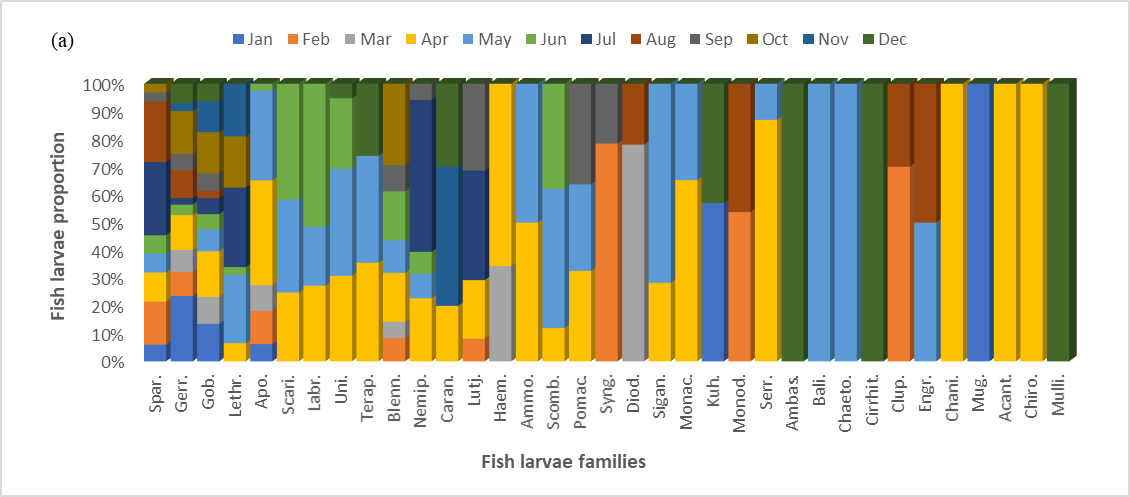


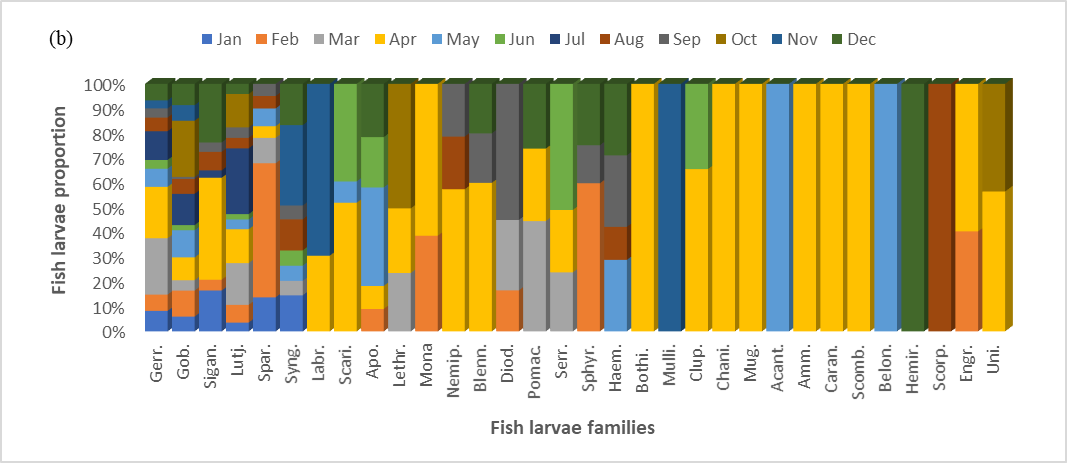


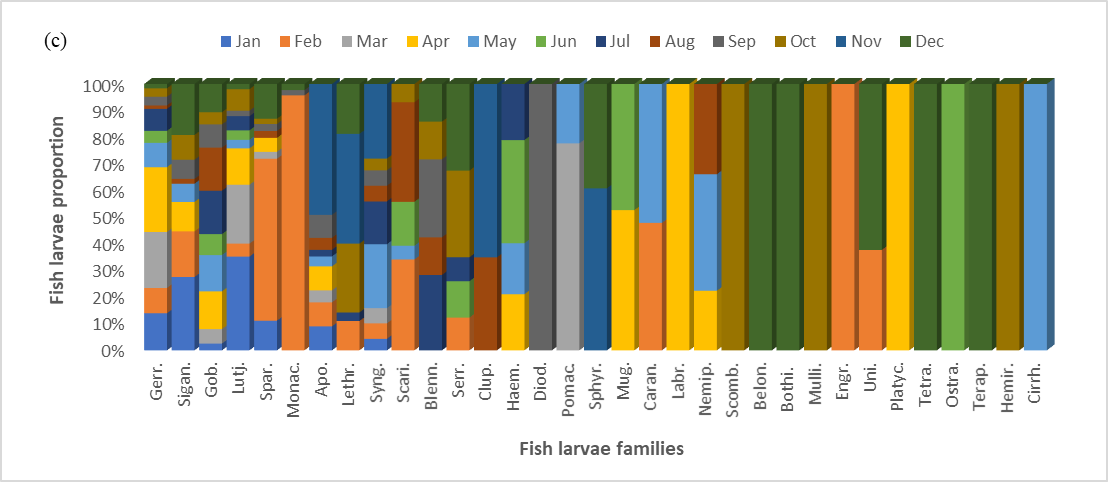


**Figure S2.** Overall mean size ± SE and monthly size (length) of individuals of fish families from the ten most abundant fish larvae families recorded in mangrove creeks (Mang), inshore seagrass meadows (InSeag) and nearshore seagrass meadows (NearSeag) within Chwaka Bay (Chwaka) and Fumba on Zanzibar (Tanzania) during the sampling period (i.e. January to December 2018).
